# Water-stable perovskite-loaded nanogels containing antioxidant property for highly sensitive and selective detection of roxithromycin in animal-derived food products

**DOI:** 10.1038/s41598-022-07030-9

**Published:** 2022-02-24

**Authors:** Jinsol Han, Mirkomil Sharipov, Soojin Hwang, Youngil Lee, Bui The Huy, Yong-Ill Lee

**Affiliations:** 1 Department of Materials Convergence and System Enginering, Changwon National University, Changwon, 51140 Republic of Korea; 2 Department of Chemistry, University of Ulsan, Ulsan, 44776 Republic of Korea; 3 Faculty of Chemical Engineering, Industrial University of Ho Chi Minh City, Ho Chi Minh City, Vietnam

**Keywords:** Chemistry, Materials science, Optics and photonics

## Abstract

Luminescent inorganic lead halide perovskite nanoparticles lack stability in aqueous solutions, limiting their application to optical sensors. Here, hybrid CsPbBr_3_-loaded MIP nanogels were developed with enhanced stability in aqueous media. Multifunctional MIP nanogels with antioxidant function and hydrophobic cavities were synthesized from HEMA derivatives in the presence of roxithromycin as a template. The CsPbBr_3_ nanoparticles were loaded into pre-synthesized MIP nanogels via in-situ synthesis with a size distribution of 200 nm. The developed CsPbBr_3_-nanogel exhibits excellent stability to air/moisture and enhanced stability toward an aqueous solvent. The developed CsPbBr_3_-loaded MIP nanogels showed a selective and sensitive detection of ROX with a limit of detection calculated to be 1.7 × 10^–5^ μg/mL (20.6 pM). The developed CsPbBr_3_-loaded MIP antioxidant-nanogels were evaluated on practical application for the quantitative determination of ROX antibiotic in animal-derived food products with excellent analytical performance. The detection of ROX in animal-derived food products showed good recovery results, making them an ideal candidate for sensing ROX.

## Introduction

Inorganic and hybrid lead halide perovskites have drawn much interest from the scientific research community owing to their outstanding electrical and optical properties (e.g., high photoluminescence quantum yield and narrow emitting bands)^1–3^. In addition, these types of materials have good compositional flexibility by various combinations of ions entering the crystal structure or by surface modification, the light emission spectral region can be easily adjusted in a large spectral wavelength region, and the preparation costs are relatively low^4,5^. Despite these excellent properties, perovskite nanoparticles have low stability to environmental factors such as temperature, pressure, solvent and have, more importantly, high sensitivity to moisture and oxygen, which hold back all sensing application that involves the use of water^6–10^. The fast dissociation and the loss of optical properties of these materials having in contact with highly polar solvents, including water, are caused by intrinsic ionic properties^11^. Other important issues of perovskite are oxidation^12^ and thermal instability^13^. Qidong Tai et al. have achieved high stability of tin-based perovskite FASnI_3_ for solar cells via the introduction of hydroxybenzene sulfonic acid or salt to prevent the oxidation of Sn^2+^ ions to Sn^2+^ upon oxygen-air exposure^14^. Although cesium lead halide perovskites are relatively less sensitive to oxygen than tin-based perovskites, they still lack stability toward oxygen^12^.

Nanogels, one type of polymeric material in which the hydrophilic function renders them capable of carrying large amounts of water molecules in their 3D networks^15–17^. During the last decades, poly(2-hydroxyethyl methacrylate) (PHEMA)-based nanogels have been widely used to develop biomedical products due to their excellent biocompatibility^18^ and dimension stability^19^. Additionally, molecularly imprinted polymer (MIP) nanogels have an excellent performance in selective binding and absorption of analytes in high concentrations due to their swelling capabilities. Various nanoparticles, including gold, silver, magnetic, and upconversion nanoparticles, have been successfully coated with MIPs for nanomedicine and sensing applications^15,20^.

Recently, the microbiome studies indicated that antibiotics present in animal-derived food cause dysregulation of gut microbiota^21,22^ and increase the resistance to antibiotics which was classed as one of the most terrible global threats of the twenty-first century^23^. Roxithromycin (ROX) is a semi-synthetic macrolide antibiotic with a 14-membered macrocyclic lactone ring and shows a 1–4 times stronger in-vivo antibacterial effect to that of erythromycin^24^. ROX has a high degree of antibacterial activity against both Gram-positive and Gram-negative bacteria^25^. ROX is rapidly absorbed, has a long half-life of elimination, and has excellent biological activity against Streptococcus, Staphylococcus aureus, Listeria, and Corynebacterium bacteria^26^. Although these advantages of ROX and other macrolides make them attractive in veterinary practice for use in the prevention and treatment of bacterial pathogens, abusive and unsafe use of macrolides antibiotics in farms can lead to the accumulation of macrolides and their residues in food products derived from animals, thereby causing allergic reactions or toxic effects on consumers^27^. Thus far, various analytical approaches for detecting antibiotics in animal-derived food products have been developed, such as rotating-disk sorptive extraction and liquid chromatography^28^, electrophoresis^29^, desorption corona beam ionization coupled mass spectrometry^30^, and microbiological assays^31^. Microbiology assays lack selectivity and sensitivity. Contrarily, mass spectrometry techniques are selective and sensitive; however, they require complicated sample pretreatment, expensive instrumentations, and well-trained personnel.

**Figure 1:**
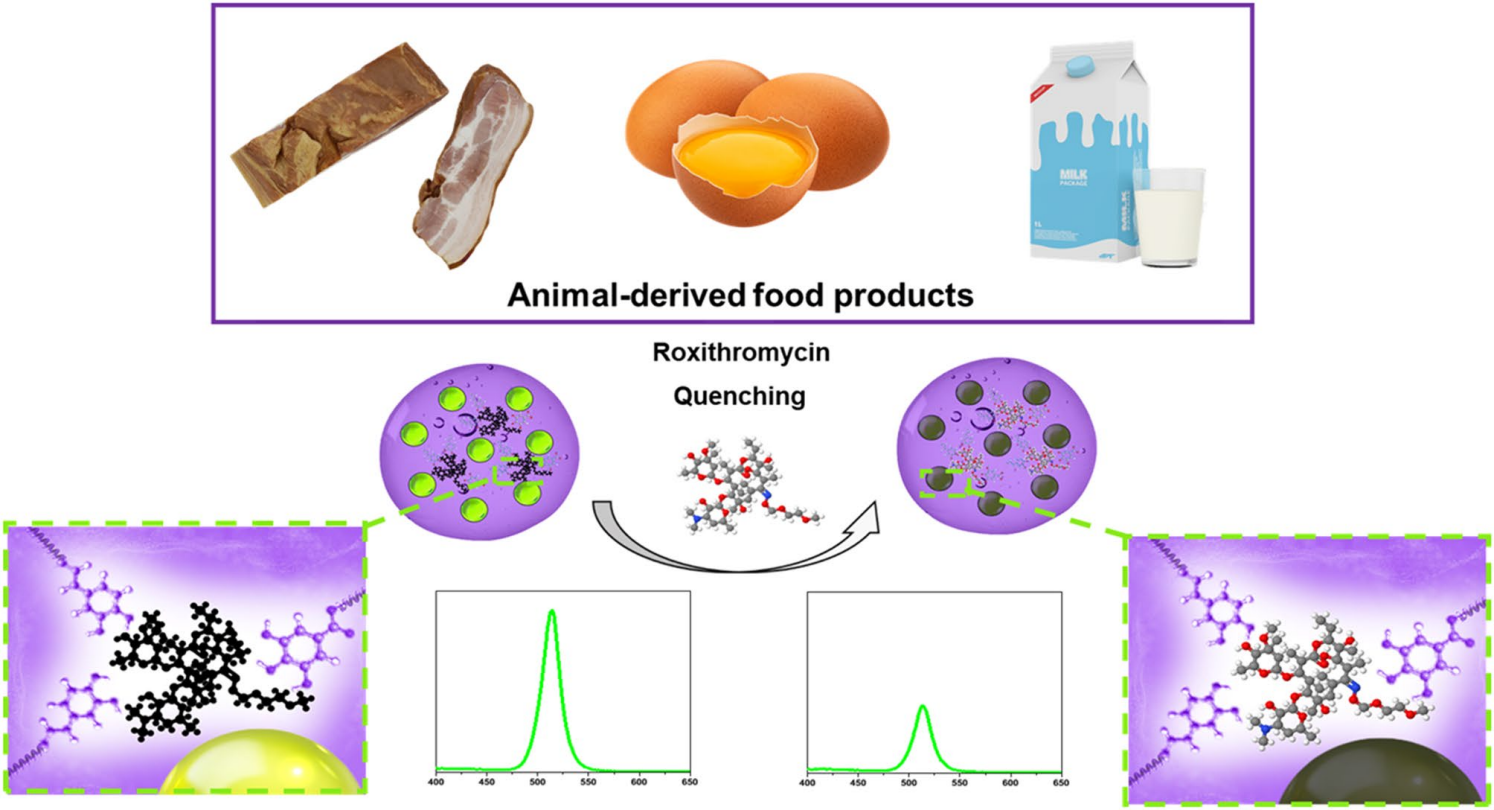
Schematic illustration of ROX sensing in animal-derived food using perovskite-loaded MIP nanogel.

Here, we have developed a novel CsPbBr_3_-loaded PHEMA MIP nanogel for the first time with high stability against water and oxidation. The method stands out due to the multifunctional MIP nanogel synthesized from four 2-(hydroxyethyl)methacrylate (HEMA) derivatives with different functions: (a) gallic-(hydroxyethyl)methacrylate ester (GA-HEMA) and caffeic-(hydroxyethyl)methacrylate ester (CA-HEMA) monomers are synthesized from naturally available gallic and caffeic acids with an antioxidant property that increases the stability of loaded perovskites toward oxygen species and oxidizing agents; (b) oleic-(hydroxyethyl)methacrylate ester (OA-HEMA) allows the formation of hydrophobic cavities that will further serve as CsPbBr_3_ perovskites reservoirs; c) polyethylene glycol-(hydroxyethyl)methacrylate ester (PEG-HEMA) increases the hydrophilicity of nanogels to reduce non-specific interactions thus resulting in higher dispersion and lower interaction with non-imprinted molecules and macromolecules. Bearing in mind that CsPbBr_3_ are sensitive to water, MIP antioxidant-nanogels were first prepared with ROX as a template, and perovskite nanoparticles were loaded into nanogels via in-situ synthesis through the hot-injection method. Developed MIP antioxidant-nanogels loaded with CsPbBr_3_ perovskite nanoparticles showed enhanced stability for water and polar solvents owing to the swelling properties of nanogel that allows the absorption of water molecules into three-dimensional networks of the polymer. Finally, the developed CsPbBr_3_-loaded MIP antioxidant-nanogels were successfully applied for the selective and sensitive detection of ROX antibiotics in animal-derived food products (Fig. 1).

## Results and discussion

### Multifunctional MIP nanogels

**Figure 2:**
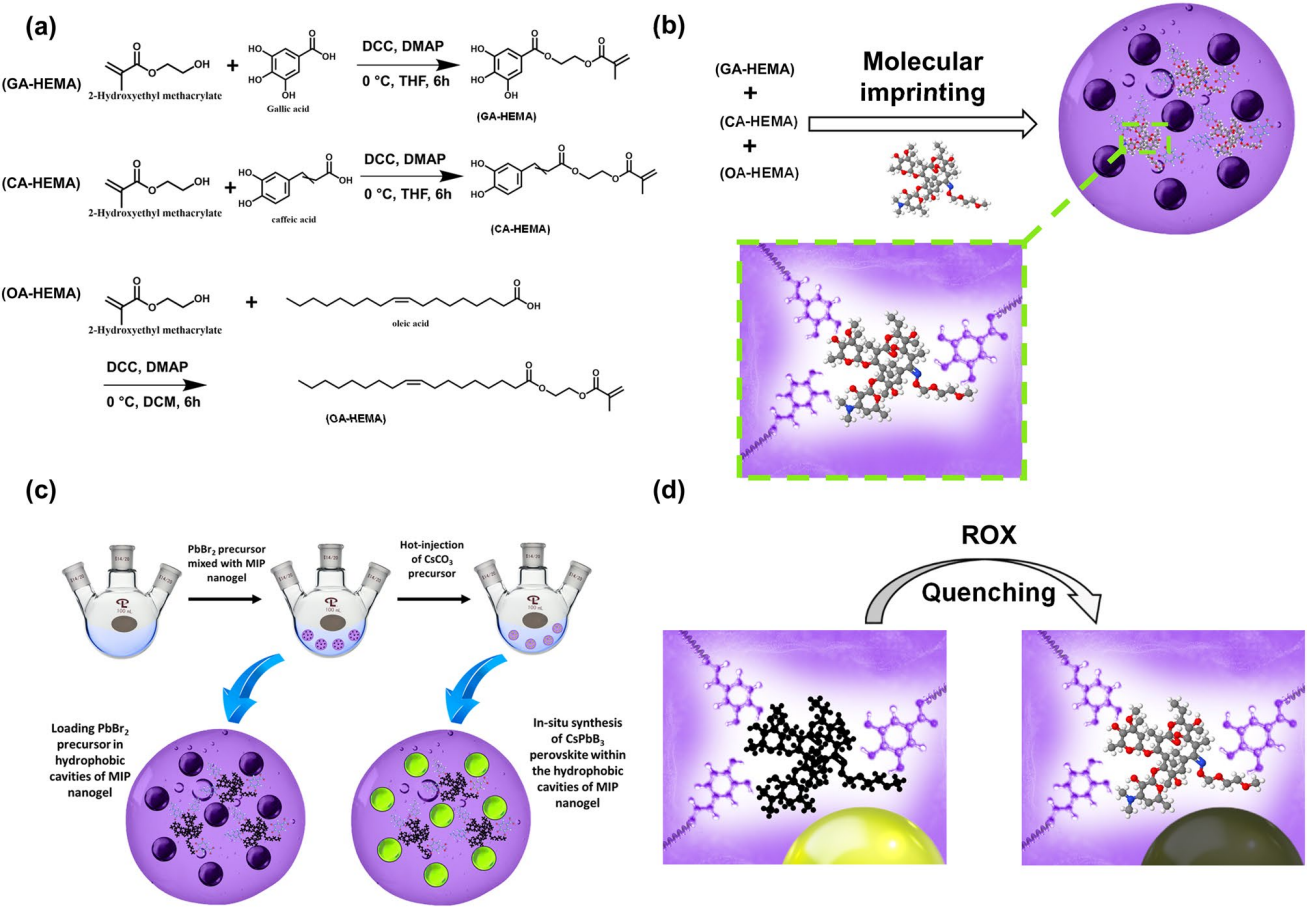
Synthesis and sensing strategies. (**a**) Synthesis of functional HEMA monomers. (**b**) Synthesis of MIP nanogels. (**c**) Loading CsPbBr_3_ perovskite nanoparticles into MIP nanogels. (d) Sensing of ROX.

**Figure 3:**
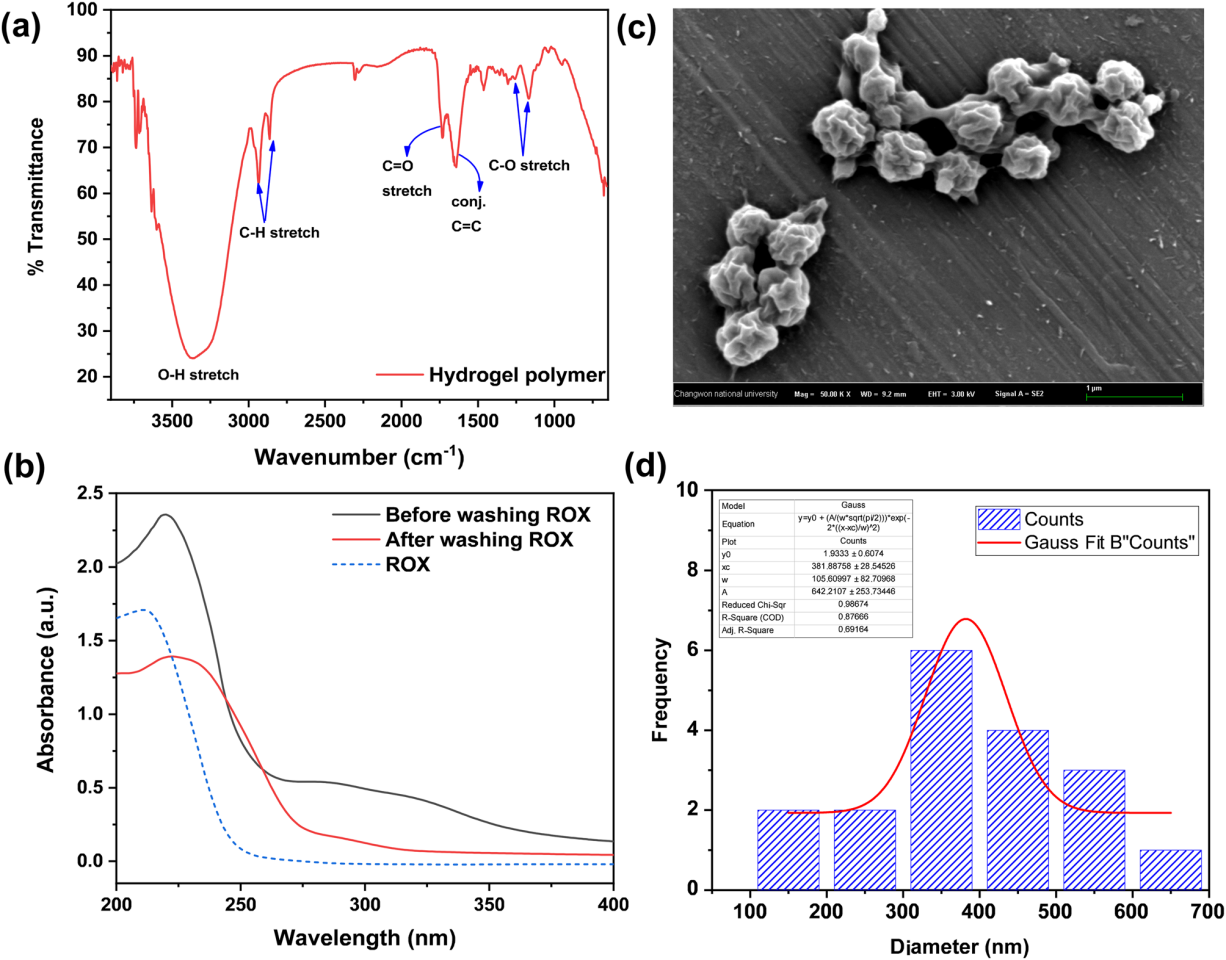
Characterization of MIP nanogels through FTIR (**a**) and UV–Vis spectrometry (**b**). LV-SEM image (**c**) and size distribution of MIP nanogels (**d**).

To develop multifunctional MIP nanogels that can enhance the stability of CsPbBr_3_ perovskites toward water and oxygen, three HEMA derivatives were synthesized. The HEMA derivatives GA-HEMA, CA-HEMA, and OA-HEMA, have been synthesized using the Steglich esterification method, as shown in Fig. 2a. GA-HEMA, CA-HEMA, and OA-HEMA were obtained as brown, yellow, and transparent oils, respectively. Three monomers were characterized by ^1^H-NMR and FT-IR techniques with the discussion presented in supporting information and Fig. S1. Multifunctional MIP nanogels were prepared via surfactant-free emulsion polymerization method using four HEMA derivatives: GA-HEMA, CA-HEMA, OA-HEMA, and PEG-HEMA in the presence of ROX as a template, as shown in Fig. 2b. Afterward, CsPbBr_3_ perovskite nanoparticles were loaded via in-situ synthesis for selective detection of ROX, as shown in Fig. 2c,d. The FT-IR spectrum of MIP nanogels showed a strong absorption peak at 1730 cm^−1^, attributed to the characteristic C=O stretch of the ester group, and a strong peak located at 1640 cm^−1^, associated with the conjugated C=C group of the aromatic groups as shown in Fig. 3a. Moreover, the two absorption peaks at 1250 cm^−1^ and 1167 cm^−1^ are attributed to the characteristic C–O stretch of ester and PEG. Finally, the relatively strong peak at 2940 cm^−1^ is caused by sp^3^ C–H stretching from oleic acid. To confirm a successful polymerization, the molecular weight of nanogel was measured via gel permeation chromatography (GPC) technique, as illustrated in Fig. S2. The average molecular weight (M_w_) of MIP nanogels was found to be 14,820 g/mol with a polydispersity (M_w_/M_n_) equal to 1.25. Whereas a non-imprinted polymer had an average M_w_ of 15,850 g/mol, and polydispersity (Mw/Mn) was 1.21. This result confirms that the presence of ROX during the polymerization step does not affect the polymerization rate, and the template was successfully removed. To confirm the successful imprinting of ROX in nanogels, UV–vis absorption was measured. As illustrated in Fig. 3b, ROX has an absorption peak located at 211 nm, which was significantly decreased after washing MIP with water several times. These results confirm the successful removal of the ROX template from MIP. The morphology and the size of MIP nanogels were studied through LV-SEM images (Figs. 3c and S3). MIP nanogels have a well-defined spherical shape with wrinkles in an unswollen state. The size distribution of nanogels was calculated to be 381 ± 105 nm (Fig. 3d).

### Perovskite-loaded MIP nanogels

Cesium lead bromide perovskite nanoparticles were loaded into MIP nanogel through in-situ synthesis by a hot-injection approach. First, MIP nanogels were dispersed in ethanol, added to the PbBr2 precursor solution, and heated at 120 °C to evaporate ethanol and distribute the PbBr_2_ precursor in nanogel cavities. In the second step, the cesium oleate precursor solution was mixed with MIP/PbBr_2_ via the hot-injection method to form CsPbBr_3_ perovskite nanoparticles loaded in hydrophobic cavities. The ratio of MIP to CsPbBr_3_ nanoparticles was investigated by fixing the concentration of CsPbBr_3_ precursors and varying the amount of MIP: 0.05, 0.1, and 0.5 g. As shown in Fig. S4, the lower concentration of MIP resulted in stronger emission due to the higher ratio of CsPbBr_3_ nanoparticles; however, their stability in water was relatively lower. The lower stability may be caused by the formation of many CsPbBr3 nanoparticles on the surface of MIP nanogels. On the other hand, the PL emission of perovskite-loaded nanogels prepared with 0.1 g of MIP nanogel showed a more significant stability enhancement. Finally, the PL emission of CsPbBr_3_-loaded MIP prepared with 0.5 g MIP has a lower initial emission but negligible improvement instability due to the efficient loading of CsPbBr_3_ nanoparticles in MIPs. It is important to note that the PL emissions in both scenarios have a slight quenching within the first 60 min confirming the formation of small quantities of unprotected CsPbBr_3_ on the surface during the hot injection method. Therefore, the optimum amount of MIP nanogel was considered 0.1 g.

**Figure 4:**
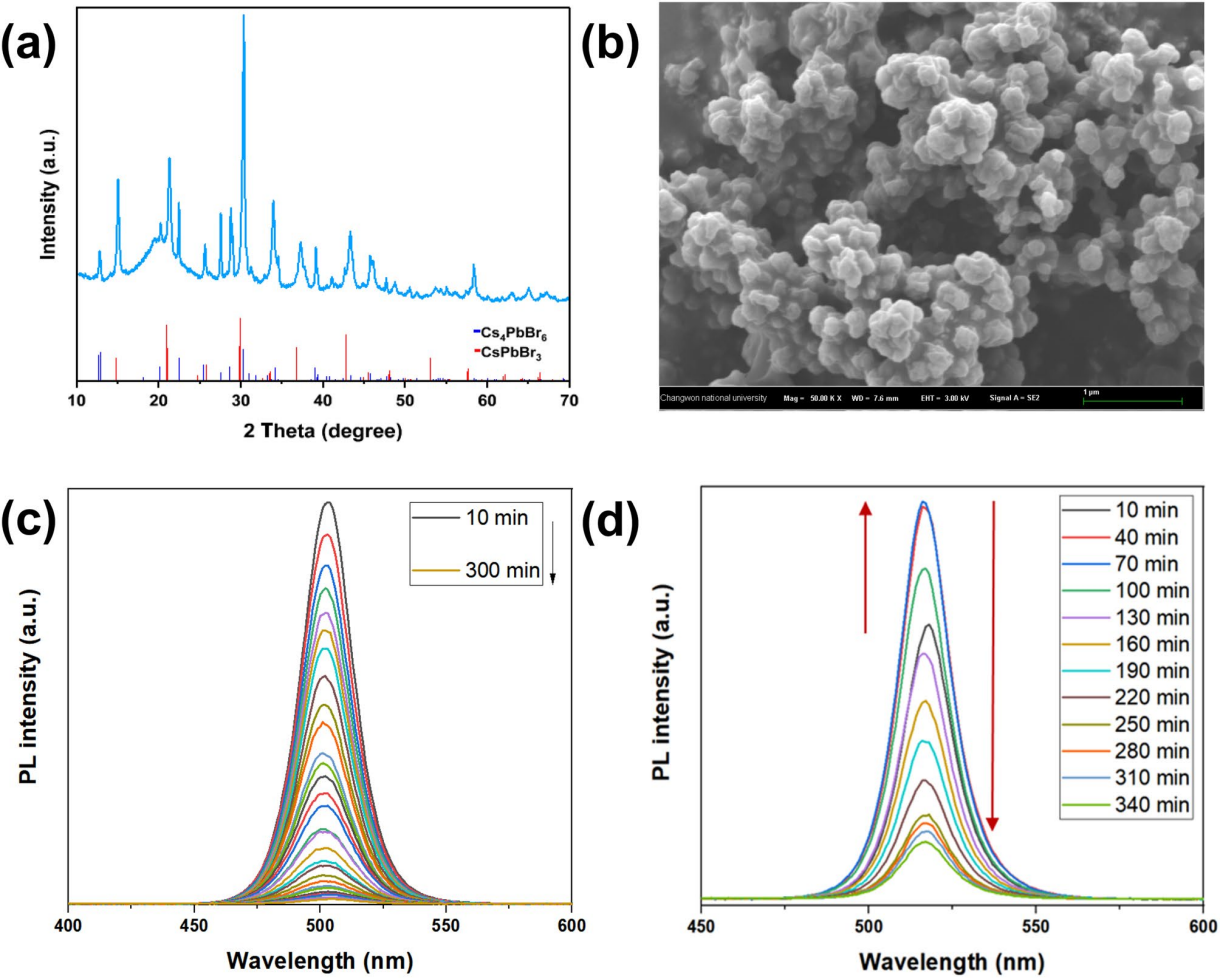
Characterization of perovskite-loaded MIP nanogels by XRD (**a**) and LV-SEM image (**b**). PL spectra of perovskite-loaded MIP nanogel in water: nanogel prepared from GA-HEMA: CA-HEMA: OA-HEMA (1 eq: 1 eq: 1 eq) (**c**) and nanogel prepared from GA-HEMA: CA-HEMA: OA-HEMA: PEG-HEMA (1 eq: 1 eq: 1 eq: 3 eq) (**d**).

To confirm the successful formation of CsPbBr_3_ perovskite nanoparticles, X-ray diffraction (XRD) analysis was performed, as shown in Fig. 4a. The synthesized perovskites have a crystal structure in which a tetragonal system is present [mp-1014168, Materials Project data repository]. The diffraction peaks at 2θ = 14.94°, 21.05°, 29.95°, 36.90°, and 42.61° are ascribed to the tetragonal CsPbBr_3_ lattices planes (110), (112), (220), (312), and (224), respectively. However, XRD patterns also indicate the transformation of the small quantity of CsPbBr_3_ into a CsBr-rich non-perovskite rhombohedral Cs_4_PbBr_6_ (JCPDS card no. 01-073-2478) phase, probably due to the accumulation of Cs ions on more accessible locations during the second step of hot-injection method. This transformation has already been observed during the synthesis with an excess of CsBr^8^. Due to the fast reaction rate, the CsCO_3_ precursor does not have enough time to spread evenly, which results in the accumulation of Cs ions in a certain location, leading to the formation of CsBr-rich non-perovskite rhombohedral Cs_4_PbBr_6_ nanoparticles. To study the morphology of MIP/CsPbBr_3_, LV-SEM analysis was performed. As shown in Fig. 4b and Fig. S5, MIP nanogels were successfully loaded with perovskite nanoparticles with a size distribution of 197 ± 71 nm, while the size of nanogels has increased to around 900 nm owing to their swelling properties. In addition, we could confirm the successful loading of CsPbBr_3_ nanoparticles into the nanogel by the presence of cesium, lead, and bromine atoms in the energy dispersive X-ray spectrum, as shown in Fig. S6. Initially, nanogels were synthesized only from CA-HEMA, GA-HEMA, and OA-HEMA monomers. However, the formed nanogels showed low water dispersibility, which further influenced the fluorescence stability of MIP/CsPbBr_3_. As shown in Fig. 4c, the loading of perovskite nanoparticles in such nanogels has increased the water stability as expected, but the absence of hydrophilic function resulted in low dispersibility and fast aggregation of MIP/CsPbBr_3_. A commercially available PEG-HEMA monomer was used as the fourth monomer to increase the dispersibility and accuracy of photoluminescence measurements. Owing to hydrophilic property and low non-specific interaction of PEG chains, PEG-HEMA enabled a better dispersibility and lower aggregation rate of nanogel. Moreover, the water stability of perovskite was further improved, and the intensity of fluorescence was increased during the first hour (Fig. 4d). A similar phenomenon was observed by Qixuan Zhong et al. after coating perovskite nanoparticles with a silica shell, which allowed better dispersion in water resulting in higher photoluminescence^32^.

### Water and oxygen stability of perovskite-loaded MIP nanogels

The water and oxygen stability of CsPbBr_3_-loaded nanogels were investigated, as shown in Fig. S7. To estimate the relative stability of developed perovskite-loaded MIP nanogels in water, the conventional CsPbBr_3_ nanoparticles prepared by hot-injection method and CsPbBr_3_-loaded nanogels were dispersed in DI water, sonicated, and PL spectra were acquired immediately, after 10 s, 1 min, and 5 min. The conventional CsPbBr_3_ nanoparticles were quenched by 75% in the first 10 s due to the decomposition of perovskite nanoparticles in presence of water molecules. On the other hand, CsPbBr_3_-loaded nanogels did not show intensity changes during the first 10 s of sonication; however, longer sonication time increased emission intensity due to the dispersion of nanogels (Fig. S7A-B)^32^. To further investigate the enhancement of the stability of CsPbBr_3_-loaded nanogels towards oxygen, we have dispersed CsPbBr_3_-loaded nanogels and conventional CsPbBr_3_ nanoparticles in toluene. Afterward, oxygen gas was bubbled for 5 min, and PL emissions were acquired. As shown in Fig. S7C-D, CsPbBr_3_-loaded nanogels have a slight decrease in intensity due to the quenching of peripheral nanoparticles, while conventional CsPbBr3 have quenched to more than 80% in 5 min. The excellent stability of MIP/CsPbBr_3_ can be explained by the insertion of perovskite nanoparticles in hydrophobic cavities of nanogel during the hot-injection method. Moreover, nanogels are composed of three-dimensional polymeric networks that absorb the water, thus lowering the contact of water molecules with perovskite nanoparticles. Finally, GA-HEMA and CA-HEMA act as an antioxidant to reduce the oxidation of perovskite by oxygen species.

### Sensitivity of ROX detection

**Figure 5:**
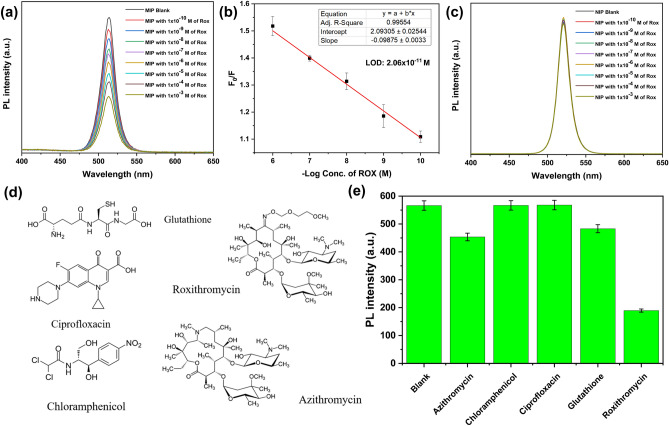
(**a**) Fluorescence emission spectra of MIP/CsPbBr_3_ in water containing different concentrations of ROX. (**b**) The calibration curve of the fluorescence intensity of MIP/CsPbBr_3_ versus ROX concentrations. (**c**) Fluorescence emission spectra of NIP/CsPbBr_3_ in water containing different concentrations of ROX. (**d**) Chemical structure of analyte tested for selectivity. Fluorescence response of MIP/CsPbBr_3_ to other analytes (**e**). All measurements were repeated three times, and standard deviations are represented as error bars.

The fluorescence response of MIP/CsPbBr3 (0.001 ppm) was studied upon the addition of increasing the concentrations of ROX between 1 × 10^–6^ and 1 × 10^–10^ M and shown in Fig. 5a. The fluorescence intensities of perovskite solution decreased gradually with an increase in ROX concentration because of the tailor-made recognition sites of the MIP/CsPbBr_3_ specific to ROX. As illustrated in Fig. 5b, the F_0_/F value represents a linear relationship with the concentration of ROX ranging from 1 × 10^–6^ M to 1 × 10^–10^ M with a good linear correlation coefficient (0.995) and low detection limit (2.06 × 10^–11^ M). The limit of detection was determined by the following equation. LOD = ${10}^{[\frac{\mathrm{log}\left(3\upsigma +{\mathrm{y}}_{0}\right)-\mathrm{a}}{b}]}$, where *a* is the intercept of fitted line, *b* is the slope of fitted line, *σ* is the standard deviation of the blank intensities of the perovskite solution, and *y*_*0*_ is the mean of blank intensities of the perovskite solution (n = 3). In order to confirm the formation of specific recognition sites to ROX within MIP nanogels, detection of the different concentrations of ROX using the perovskite-loaded non-imprinted polymer (NIP) nanogels was performed. In the case of NIP, a slight change in the fluorescence intensity of perovskite has been observed, which is explained by minor quenching of more accessible perovskite particles located near the surface by ROX. However, compared to the MIP, the changes in fluorescence intensity were insignificant according to the concentration of the ROX (Fig. 5c). These results demonstrate that the developed MIP/CsPbBr_3_ particles have great properties to detect ROX with high sensitivity.

### Selectivity of ROX detection

Selectivity tests were performed to evaluate whether the developed MIP/perovskite can selectively detect only ROX among various antibiotics and common tripeptide Glutathione (Fig. 5d). Four different antibiotics (Azithromycin, Chloramphenicol, Ciprofloxacin, and ROX) were selected as analytes and prepared in ethanol/water at a concentration of 1 mM (Fig. 5d). As shown in Fig. 5e, non-macrolide antibiotics, such as chloramphenicol and ciprofloxacin, did not affect the fluorescence intensity of the developed sensor, while azithromycin, which has structural similarities to ROX, induced a slight decrease in fluorescence intensity. Bearing in mind that this sensor has a potential in the analysis of animal-derived food products that contain different proteins, the selectivity to common tripeptide glutathione was investigated. A slight quenching of MIP/CsPbBr_3_ observed in the presence of glutathione can be explained by the transformation of CsPbBr_3_ into a non-luminescent phase. A similar phenomenon was observed in previously reported work, where CsPbBr_3_ was transformed into non-luminescent Cs_4_PbBr_6_ in the presence of thiol-alkyl and residual oleylamine^33^. In contrast to tested molecules, ROX exhibited significant quenching efficiency more than twofold, thus confirming the efficient selectivity of the developed sensor towards ROX.

### Mechanism of ROX detection

**Figure 6:**
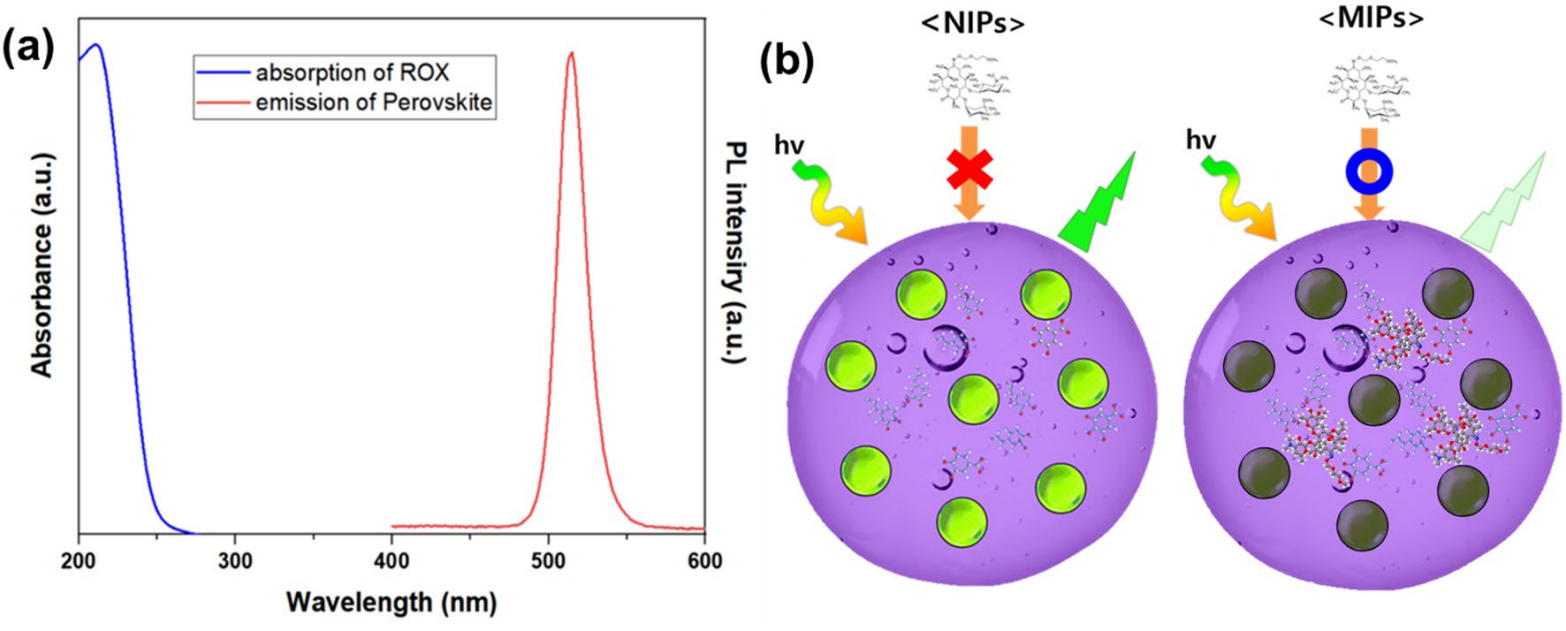
(**a**) PL and UV spectra of the emission of perovskite and absorption of ROX, (**b**) Schematic illustration of the proposed quenching mechanism of MIP/CsPbBr_3_ during the exposure to ROX.

Different quenching mechanisms have been considered, including molecular interactions by electrostatic or hydrogen bonding between analyte and perovskite, Förster resonance energy transfer (FRET), inner filter effect (IFE), and perovskite phase transformation or oxidation. In the FRET mechanism, the collision during dynamic quenching between the fluorescent material in an excited state and the quencher molecule results in energy loss and return to the ground state. Moreover, it requires that the emission spectrum of the energy donor must overlap with the absorption spectrum of the energy acceptor. The synthesized MIP/CsPbBr_3_ have a typical peak emission of cesium lead bromide at 520 nm, whereas the ROX absorption peak is at 221 nm, as shown in Fig. 6a. These results suggest the absence of spectral overlap between perovskite and ROX; therefore, the probability of energy transfer in the fluorescence quenching mechanism is minuscule. Thus, possible quenching mechanisms are either phase transformation or the decomposition of perovskite by ROX. In the case of NIP to which ROX is not added as a template molecule in the polymerization process, no cavity complementary to ROX exists in the polymer. Therefore, ROX is added during the detection process cannot bind the NIP; thus, the fluorescence emission of perovskite is not affected. However, in the case of MIP, cavities complementary to ROX are formed during the polymerization step. Thereby, during the detection process, ROX binds to the cavity in the polymer, and structural decomposition of perovskite is induced by the N-oxime functional group having the oxidative property of ROX (Fig. 6b). To further confirm our hypothesis, perovskite-loaded MIP nanogels before and after ROX treatment were analyzed via X-ray photoelectron spectroscopy (XPS). As shown in Fig. S8, the Pb 4f. peaks of perovskite-loaded MIP nanogels at 137.30 and 142.20 eV corresponded to Pb 4f_7/2_ and Pb 4f_5/2_, respectively. As reported in previous work, the stoichiometric reaction for CsPbBr_3_ can be written as follows: 2CsPbBr_3_·H_2_O + O_2_ + CO_2_ = 2CsBr + PbCO_3_ + Pb(OH)_2_ + 2HBr + Br_2_; Pb(OH)_2_ = PbO + H_2_O^34^. The XPS analysis showed that perovskite-loaded MIP nanogels treated with ROX undergo a decomposition process, thus resulting in peaks shift to higher binding energies. The peaks appeared at 137.72 and 142.64 eV corresponds to the formed PbCO_3_^34^.

### Practical application of the sensor for animal-derived food products

The practical relevance of perovskite-loaded MIP nanogels for detecting ROX was investigated in three animal derived-food products: meat, milk, and egg. Pork meat, eggs, and milk products were acquired from local grocery stores in Changwon, Republic of Korea. Prior analysis, samples were extracted by experimental procedure reported in previous works and spiked with ROX standard solution^35^. Acceptable recoveries and relative standard deviations (RSDs) of ROX spiked in milk, porcine muscle, and egg samples using MIP/CsPbBr_3_ have been achieved, as shown in Table 1. The recoveries of ROX ranged from 99.2 ~ 100%, 101 ~ 102%, and 98.2 ~ 99.1%, with RSDs, ranged from 6.32 ~ 11.7%, 3.66 ~ 6.99%, and 5.84 ~ 6.46% for milk, pork, and eggs, respectively. These findings demonstrate the accuracy of the developed sensor for selective and sensitive detection of ROX in animal-derived food samples, thus revealing the excellent potential for practical application. The analytical performances of the synthesized MIP/CsPbBr_3_ for detecting ROX were compared to several methods previously reported. As shown in Table 2, most reported methods are time-consuming and require bulky instruments with well-trained technicians. By contrast, the fluorescence sensor reported in this work does not require expensive instruments and long analysis time. Moreover, this sensor has shown good analytical performance with a wide dynamic range from 8.4 × 10^–5^ to 8.4 × 10^–1^ μg/mL and a lower detection limit of 1.7 × 10^–5^ μg/mL (20.6 pM) compared with those in the previously reported sensing methods.

**Table 1:** Results of ROX detection in milk, pork, and eggs real samples by developed MIP/perovskites.

Samples	Added (× 10^–5^ M)	Found (× 10^–5^ M)	Recovery (%)	RSD (n = 3, %)
Milk	1.00 × 10^–3^	0.99 ± 0.09 × 10^–3^	99.3	9.32
	1.00 × 10^–1^	0.99 ± 0.11 × 10^–1^	99.2	11.7
	1.00 × 10	1.00 ± 0.06 × 10	100	6.32
Pork	1.00 × 10^–3^	1.02 ± 0.03 × 10^–3^	102	3.66
	1.00 × 10^–1^	1.03 ± 0.07 × 10^–1^	102	6.99
	1.00 × 10	1.01 ± 0.04 × 10	101	4.72
Eggs	1.00 × 10^–3^	0.99 ± 0.06 × 10^–3^	99.1	6.46
	1.00 × 10^–1^	0.99 ± 0.06 × 10^–1^	98.7	6.07
	1.00 × 10	0.98 ± 0.05 × 10	98.2	5.84

**Table 2:** Comparison of CsPbBr_3_-loaded MIP nanogels with other general methods for the detection of ROX.

	Analytical technique	Linear Range (μg/mL)	LOD (μg/mL)	References
1	High-performance liquid chromatography (HPLC)	0.05–20.0	5.0 × 10^−2^	^36^
2	Electrochemistry (EC)	4.2–84	4.0 × 10^−1^	^37^
3	Fluorescence using CdTe quantum dots (FL)	25.0–350.0	4.6	^38^
4	Aqueous two-phase system extraction (ATPSE)	1.0–20.0	3.0 × 10^−2^	^39^
5	Capillary electrophoresis (CE)	0.02–201.0	7.0 × 10^−3^	^29^
6	Fluorescence using MIP/CsPbBr_3_ (FL)	8.4 × 10^–5^–8.4 × 10^–1^	1.7 × 10^–5^	This Study

## Conclusion

In conclusion, CsPbBr_3_-loaded MIP nanogel with water and oxygen stability was developed via in-situ synthesis of cesium lead bromide. Multifunctional MIP nanogel with antioxidant properties showed enhanced stability of CsPbBr_3_ nanoparticles in water and oxygen. MIP nanogels showed highly sensitive and selective detection of imprinted macrolide ROX with a wide dynamic range and a low limit of detection calculated to be 20.6 pM. Moreover, the detection of ROX via the developed sensor in animal-derived food products showed its high potential for practical application with good recoveries and acceptable RSDs. We believe that the achieved stability of CsPbBr_3_-loaded nanogels will also inspire researchers to develop new hybrid nanomaterials for other applications such as solar cell and LEDs.

## Methods

### Materials and characterization methods

The chemicals and characterization methods are described in supporting information.

### Esterification of gallic, caffeic, and oleic acid

#### GA-HEMA

Monomers were synthesized via Steglich esterification method with minor modifications^40^. Briefly, to the precooled solution at 0 °C containing gallic acid (1.00 g, 5.88 mmol, 1 eq) and HEMA (1.53 g, 11.8 mmol, 2 eq) in tetrahydrofuran (THF) (40 mL), an activator N,N′-dicyclohexylcarbodiimide (DCC) was added dropwise (1.21 g, 5.88 mmol, 1 eq) in THF (10 mL). The mixture was continuously stirred for 30 min at 0 °C, then added 4-(N,N-dimethylamino)-pyridine (DMAP) catalyst (0.0718 g, 0.59 mmol, 0.1 eq), and stirred again for next 24 h at 0 °C, then allowed to reach room temperature. The precipitate was filtered out when the reaction was finished to remove N,N’-dicyclohexylurea (DCU), and the substance was concentrated under reduced pressure. The oily product was poured into a mixture of chloroform and isopropanol (CHCl_3_:C_3_H_8_O = 3:1, v/v). To remove the catalyst and DCU residues, the organic layer was repeatedly washed with 1 M HCl solution, then with saturated aqueous sodium bicarbonate solution, with brine solution, and finally with deionized water. The solvent was removed under reduced pressure, and the product was further purified by adding cold diethyl ether (0 °C) to filter out catalysts with poor solubility in the cold solvent. The purified compound was analyzed using a proton nuclear magnetic resonance (^1^H NMR) spectrometer and Fourier transform infrared (FT-IR) spectrometer.

#### CA-HEMA

CA-HEMA was synthesized through the same experimental protocol with minor changes in purification steps. After removing catalyst and DCU residual by washing step, the organic layer was concentrated under reduced pressure, and the product was further purified by adding cold acetone (0 °C) to filter out catalysts with poor solubility in the cold acetone solvent. CA-HEMA was characterized via ^1^H NMR and FT-IR.

#### OA-HEMA

OA-HEMA was synthesized via the same experimental procedure used to synthesize GA-HEMA with a minor modification in the synthesis step. The solvent THF was replaced by dichloromethane (DCM) due to the better solubility of reactants in DCM. Briefly, To the precooled solution at 0 °C, containing oleic acid (1.66 g, 5.88 mmol, 1 eq) and HEMA (1.53 g, 11.8 mmol, 2 eq) in DCM (40 mL), activator DCC was added dropwise (1.21 g, 5.88 mol, 1 eq) in DCM (10 mL). The mixture was continuously stirred for 30 min at 0 °C, then added DMAP catalyst (0.0718 g, 0. 59 mmol, 0.1 eq), and stirred again for the next 24 h at 0 °C and allowed to reach room temperature. The product was purified and characterized through the same procedures and techniques described in previous sections.

### Synthesis of antioxidant MIP nanogels

Nanogels were synthesized via the surfactant-free emulsion polymerization method reported previously with minor modifications^41^. The poly(vinyl alcohol) (0.5 g), was fully dissolved in 50 ml water/THF solvent (4:1, v/v) to form a continuous phase. Then, the mixture of GA-HEMA, CA-HEMA, OA-HEMA, PEG-HEMA, and EGDMA (0.82 mmol, 0.82 mmol, 0.82 mmol, 2.46 mmol, and 0.05 mmol, respectively) were added to the dispersion under ultrasonication and kept for an additional half an hour. In the case of MIP, 1 mmol ROX (template) was dissolved in the solution. Ammonium persulphate was used as an initiator with a concentration of 0.44 mg/ml in the monomer phase. Firstly, the initiator was added to the reaction mixture and nitrogen gas was bubbled for approximately 1–2 min under stirring to remove dissolved oxygen. Afterward, the solution was stirred at 70 °C for 24 h under a nitrogen atmosphere. Upon the completion of polymerization, nanogels were collected by centrifugation at 15,000 rpm for 30 min and resuspended in ethanol/water (1:1). This procedure was repeated several times to eliminate the unreacted monomer, initiator, and template analyte.

### Loading CsPbBr_3_ perovskites in MIP nanogels by the hot-injection method

Firstly, cesium oleate was prepared by stirring cesium carbonate (0.25 mmol) and 0.25 mL of oleic acid in 4 mL of 1-octadecene (ODE) under nitrogen gas at 120 °C for 1 h in a 3-neck flask. Separately, lead(II) oxide (0.188 mmol), ammonium bromide (0.585 mmol), 0.5 mL of oleic acid, and 0.5 mL of oleylamine as a capping ligand are added in 5 mL of ODE under nitrogen gas to fully dissolve the lead halide. The temperature is raised and kept at 120 °C for 30 min in vacuo. When this solution becomes transparent, 0.1 g of MIP nanogel in 1 mL of ethanol is injected. Afterward, the mixture is heated to evaporate ethanol for 30 min. Afterward, the cesium oleate solution at 120 °C is swiftly injected, and the mixture is stirred for 5–10 s before quenching the reaction in an ice-water bath (0 °C). MIP nanogel loaded with perovskite nanoparticles was poured in ethyl acetate and collected by centrifugation at 10,000 rpm for 10 min.

## Supplementary Information

Supplementary Information.

## Data Availability

The datasets used and/or analysed during the current study are available from the corresponding author on reasonable request.

## Abbreviations

^1^H NMR: Proton nuclear magnetic resonance
CA-HEMA: Caffeic-(hydroxyethyl)methacrylate ester
DCC: *N*,*N*′-Dicyclohexylcarbodiimide
DCM: Dichloromethane
DCU: *N*,*N*′-Dicyclohexylurea
DMAP: 4-(*N*,*N*-dimethylamino)-pyridine
FRET: Förster resonance energy transfer
FT-IR: Fourier transform infrared
GA-HEMA: Gallic-(hydroxyethyl)methacrylate ester
GPC: Gel permeation chromatography
HEMA: (2-Hydroxyethyl methacrylate)
IFE: Inner filter effect
MIP: Molecularly imprinted polymer
MIP/CsPbBr3: Cesium lead bromide perovskite-loaded MIP nanogels
NIP: Non-imprinted polymer
OA-HEMA: Oleic-(hydroxyethyl)methacrylate ester
ODE: 1-Octadecene
PEG-HEMA: Polyethylene glycol-(hydroxyethyl)methacrylate ester
PHEMA: Poly(2-hydroxyethyl methacrylate)
ROX: Roxithromycin
THF: Tetrahydrofuran
XRD: X-ray diffraction
