# Supplementary material for: Water-stable perovskite-loaded nanogels containing antioxidant property for highly sensitive and selective detection of roxithromycin in animal-derived food products
Source: Sci Rep. 2022 Feb 24;12:3147. doi: 10.1038/s41598-022-07030-9 (PMC8873197; doi:10.1038/s41598-022-07030-9)
Supplement: Supplementary file 1 — Supplementary Information. [file 41598_2022_7030_MOESM1_ESM.docx]

Supporting Information

**Water-stable Perovskite-loaded Nanogels Containing Antioxidant Property for Highly Sensitive and Selective Detection of Roxithromycin in Animal-derived Food Products**

Jinsol Han^1^, Mirkomil Sharipov^1,2^, Soojin Hwang^1^, Youngil Lee^2^*, Bui The Huy^1^,

Yong-Ill Lee^1^*

^1^Department of Materials Convergence and System Engineering, Changwon National University, Changwon 51140, Republic of Korea

^2^Department of Chemistry, University of Ulsan, Ulsan 44776, Republic of Korea

*Corresponding Author; E-mail: yilee@changwon.ac.kr (Y.-I. Lee), nmryil@ulsan.ac.kr

(Y. Lee)

**Keywords**: MIP nanogel, water-stable perovskite, animal-derived food, antibiotics, roxithromycin.

## 1. Materials and characterization methods

1.1. Materials

The following materials: gallic acid (97.5-102.5%), oleic acid (90%), 2-hydroxyethyl methacrylate (HEMA, 97%), N,N′-dicyclohexylcarbodiimide (DCC, 99%), 4-(N,N-dimethylamino)-pyridine (DMAP, 99%), poly(vinyl alcohol) (99%), ethylene glycol dimethacrylate (EGDMA), lead(Ⅱ) oxide (99.999%), oleylamine (70%), 1-octadecene (90%), and caffeic acid (98%) were purchased from Sigma-Aldrich (South Korea). Dichloromethane (HPLC grade) and cesium carbonate (99.9%) were purchased from Alfa Aesar (South Korea). Roxithromycin (ROX, >95%, HPLC grade) and polyethylene glycol monomethyl ether methacrylate (PEG-HEMA) were purchased from TCI chemicals. Ammonium persulfate (95%) and tetrahydrofuran (HPLC grade) were purchased from Duksan reagents. Ammonium bromide (99%) were purchased from Acros organics. Sodium hydrogen carbonate, hydrochloric acid (36%), chloroform (HPLC grade) and 2-propanol (HPLC grade) were purchased from Daejung chemicals. Sodium chloride (99%) and acetone (99.5%) were purchased from Samchun chemicals. All chemicals and reagents were used directly without further purification. In all experimental procedures deionized water of Milli-Q grade was used.

1.2. Characterization

The Fourier transform infrared spectra (FT-IR) were recorded on an FT/IR-6300 Fourier Transform Infrared Spectrometer (Jasco, Japan). ^1^H-NMR spectra of the synthesized monomers in DMSO were measured with a Bruker ux NMR 400 MHz instrument. Gel permeation chromatography (GPC) was conducted using Waters 2414 Refractive Index Detector. The fluorescence intensity measurements were carried out using an FP-6500 spectrofluorometer (Jasco, Japan) with a quartz cuvette of 1cm path-length. Field-emission scanning electron microscopy (FE-SEM) measurements were recorded by using a CZ/MIRA LMH FE-SEM microscope. The absorption spectra were obtained by using a spectrophotometer, Agilent 8543 UV/Vis (Agilent, USA). Centrifuge were carried out using a LaboGene 1580R centrifugal separator (LaboGene, Korea). X-ray diffraction (XRD) was carried out on a Bruker D8 Discover diffractometer with Cu Kα radiation (λ = 0.1542 nm). Samples were fixed to a glass substrate and analyzed.

1.3. GA-HEMA: 2-((1-(3,4,5-trihydroxyphenyl)vinyl)oxy)ethyl methacrylate

GA-HEMA was obtained in form of a brown oil by Steglich esterification. The obtained product was characterized by ^1^H-NMR and FT-IR. The proton of carboxylic acid has disappeared and a new peak at 4.10 ppm has appeared which corresponds to the formation of the ester group. At 6.94 ppm, that peak was derived from the benzene ring of gallic acid. The proton peaks of hydroxyl groups at the phenol position don’t appear in the NMR results because they are labiles (**Figure S1**). ^1^H-NMR (400 MHz, in DMSO, 298 K, Figure) δ= 6.94 (s, 1H, gallic acid moiety), 6.06 (dt, 1H, -CH2), 5.68 (p, 1H, -CH2), 4.10 (m, 2H, -CH2), 3.61 (m, 2H, -CH2), 1.89 (s, 3H, -CH3). In the FTIR spectrum of ester, the strong absorption bands at 1700 cm^−1^, assigned to the characteristic C=O stretch of the ester group and indicated a strong sharp band about at 1630 cm^−1^ conjugated C=C group of the benzene ring. Also, the strong two absorption peaks at 1300 and 1164 cm^-1^ assigned to the characteristic C-O stretch of ester. The band at 2920 cm^-1^ is attributed to sp3 C-H stretch (**Figure S1**). These results confirm the successful synthesis and good purity of GA-HEMA.

1.4. CA-HEMA: (E)-2-((3-methylbuta-1,3-dien-2-yl)oxy)ethyl 3-(3,4-dihydroxyphenyl)acrylate

CA-HEMA monomer (CA-HEMA) was synthesized through the same method as GA-HEMA and the product after purification was in form of a yellow oil. The product was characterized by ^1^H-NMR and FT-IR. The proton of carboxylic acid has disappeared and a new peak at 4.10 ppm has appeared which corresponds to the formation of the ester group. At 6.79 ppm, that peak was derived from the benzene ring of caffeic acid. Also, peaks located at 9.69 ppm and 9.20 ppm are attributed to the phenol protons of caffeic acid (**Figure S1**). ^1^H-NMR (400 MHz, in DMSO, 298 K, Figure) δ= 9.69 (s, 1H, caffeic acid moiety), 9.20 (s, 1H, caffeic acid moiety), 7.64 (m, 1H, =CH), 7.09 (m, 1H, caffeic acid moiety), 6.79 (m, 1H, caffeic acid moiety), 6.52 (m, 1H, -=CH), 6.06 (m, 1H, =CH_2_), 5.68 (m, 1H, =CH_2_), 4.10 (m, 2H, -CH_2_), 1.88 (d, 3H, -CH_3_). In the FTIR spectrum of ester, the strong absorption bands at 1705 cm^−1^, assigned to the characteristic C=O stretch of the ester group and indicated a strong band about at 1630 cm^−1^ conjugated C=C group of the benzene ring. Also, the strong two absorption peaks at 1300 and 1164 cm^-1^ assigned to the characteristic C-O stretch of ester. The band at 2940 cm^-1^ is caused by sp^3^ C-H stretch (**Figure S1**). These results confirm that CA-HEMA monomer was successfully synthesized and purified.

**1.5. OA-HEMA: 2-(methacryloyloxy)ethyl oleate**

The Steglich esterification of oleic acid and HEMA gave a product in form of colorless oil which was further characterized by ^1^H-NMR and FT-IR. The proton of carboxylic acid has disappeared and a new peak at 4.28 ppm has appeared which corresponds to the formation of the ester group (**Figure S1**). ^1^H-NMR (400 MHz, in DMSO, 298 K, Figure) δ= 6.03 (m, 1H, =CH_2_), 5.69 (h, 1H, =CH_2_), 5.32 (m, 2H, =CH), 4.28 (m, 4H, -CH_2_), 2.29 (t, 2H, -CH_2_), 1.98 (qt, 4H, -CH_2_), 1.88 (dt, 2H, -CH_2_), 1.50 (p, 2H, -CH_2_), 1.24 (m, 20H, -CH_2_), 0.85 (m, 3H, -CH_3_). The FTIR spectrum of ester showed a strong absorption bands at 1730 cm^−1^ which is assigned to the characteristic C=O stretch of the ester group. Also, the strong two absorption peaks at 1300 and 1155 cm^-1^ assigned to the characteristic C-O stretch of ester (**Figure S1**).

**
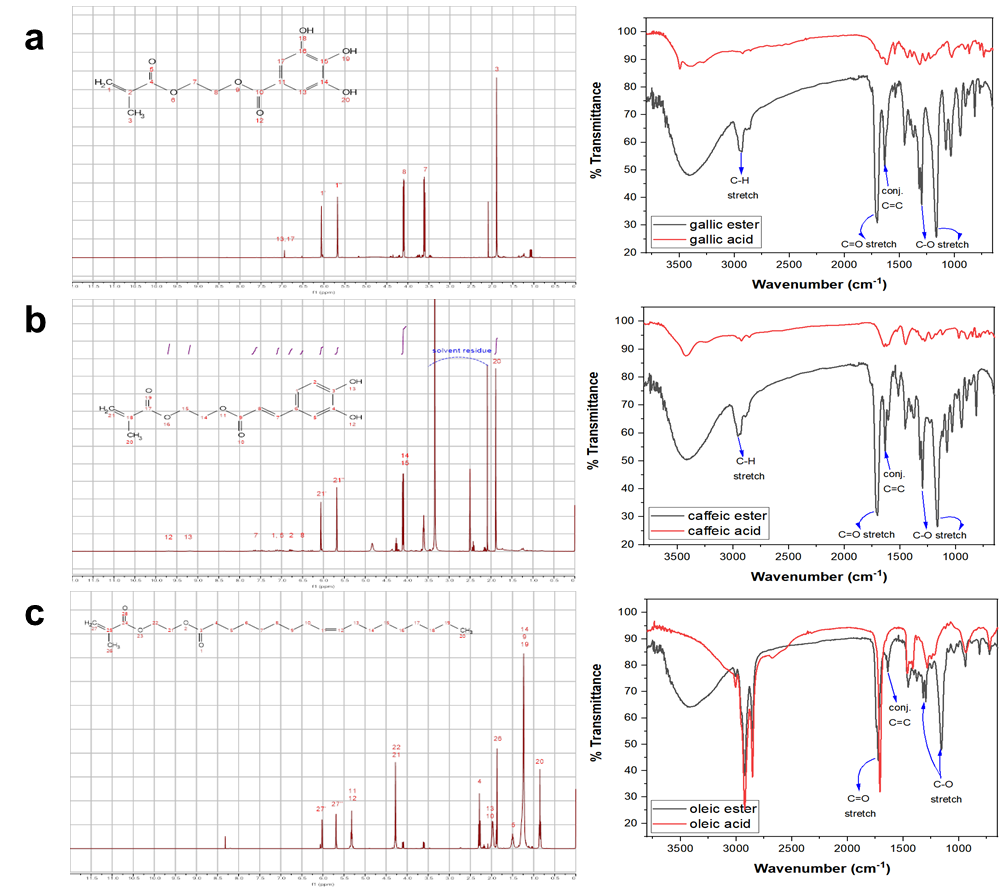
**

**Figure S1.** ^1^H-NMR spectra (400 MHz, in DMSO, 298 K) and FTIR spectra of GA-HEMA (a), CA-HEMA (b), and OA-HEMA (c).


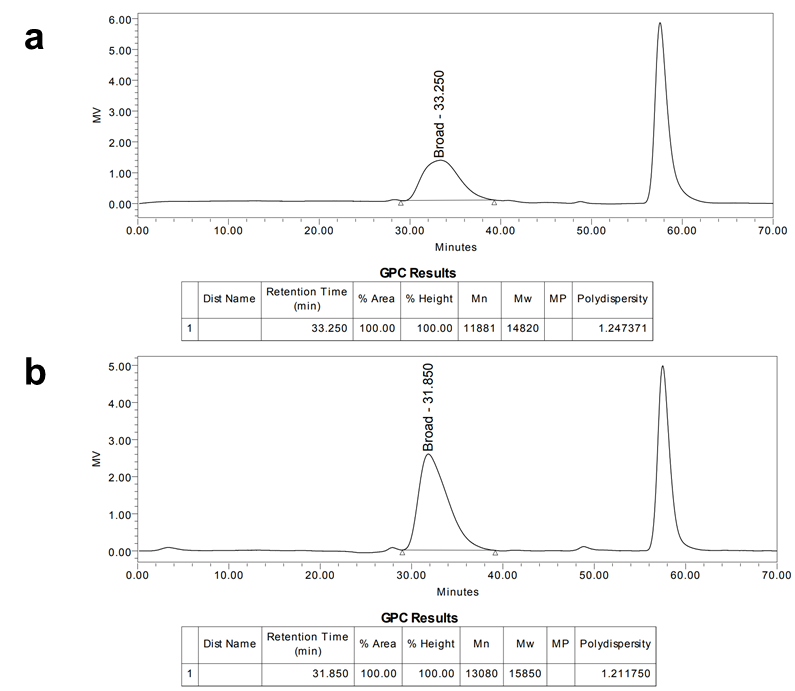


**Figure S2.** Gel Permeation Chromatogram (GPC) of MIPs and NIPs (Mn: Number-average molecular weight, Mw: Weight-average molecular weight).


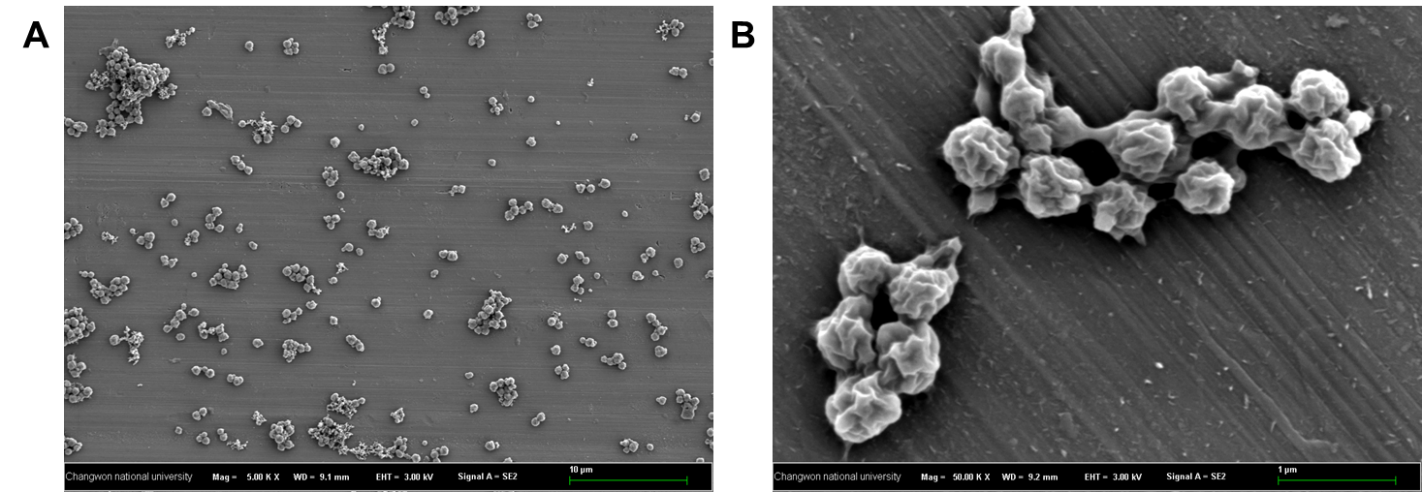


**Figure S3**. LV-SEM image of nanogel at 5 kX magnification (A) and 50 kX magnification (B)


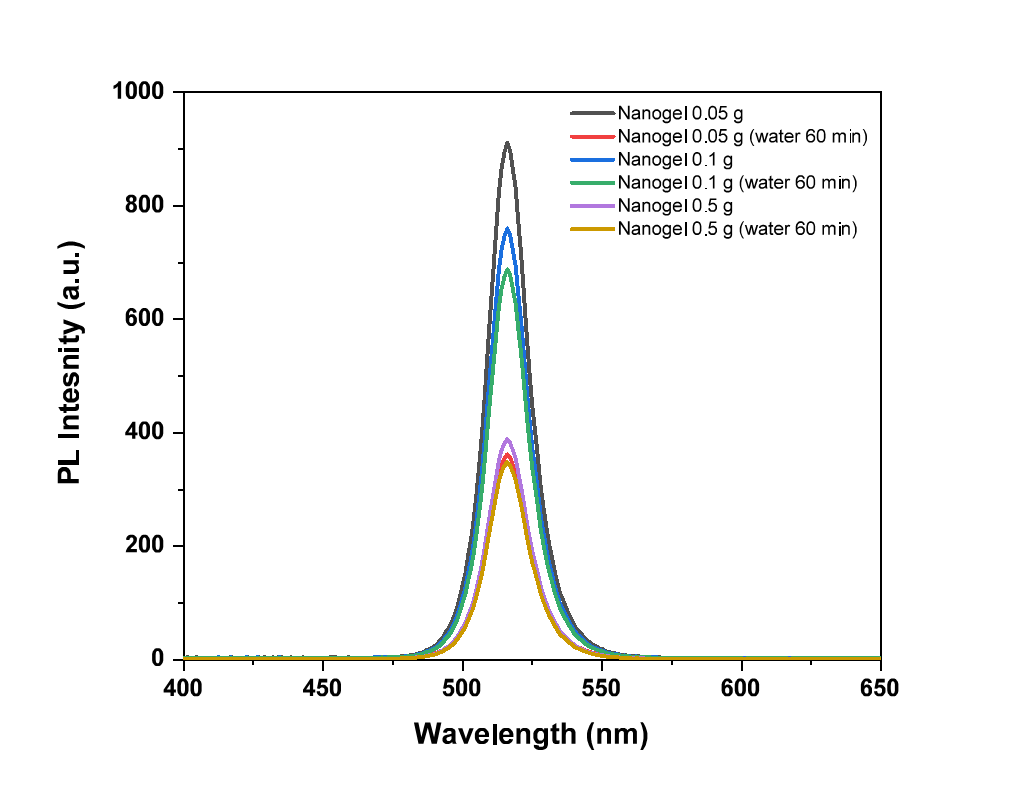


**Figure S4.**PL emission spectra of CsPbBr_3_-loaded MIP nanogels prepared with three different amount of MIP nanogels: 0.05 g, 0.1 g, and 0.5 g.


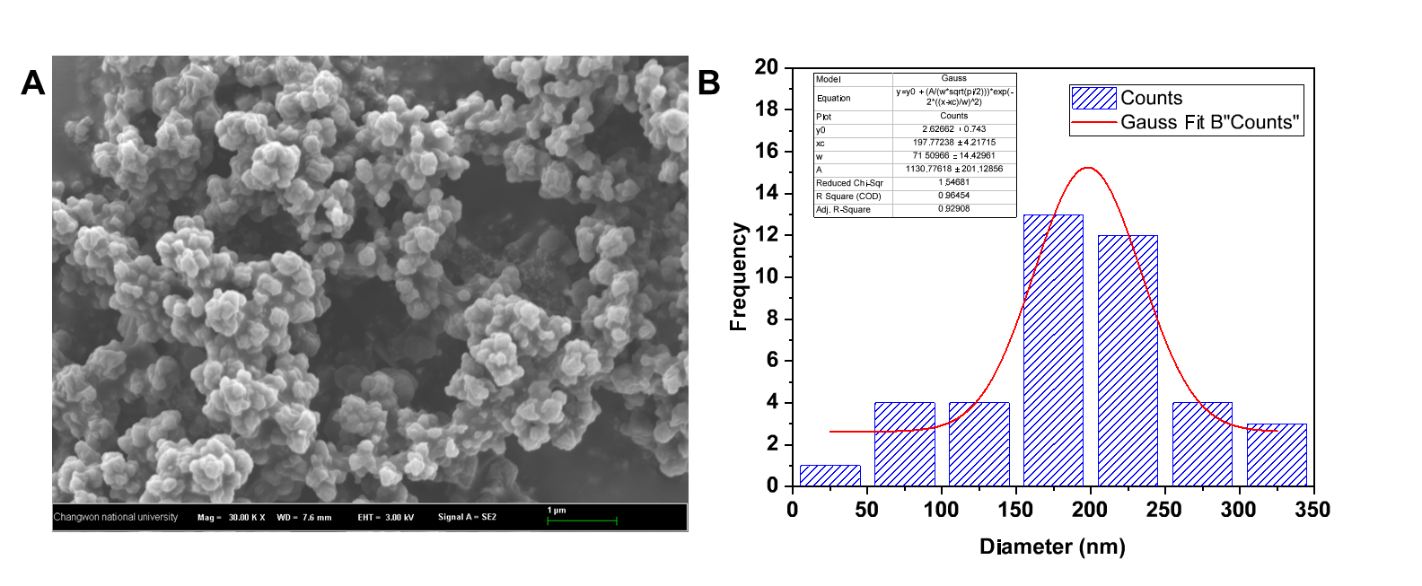


**Figure S5.**LV-SEM image of CsPbBr_3_ perovskite-loaded MIP nanogel (A) and size distribution of CsPbBr_3_ perovskite (B)

**
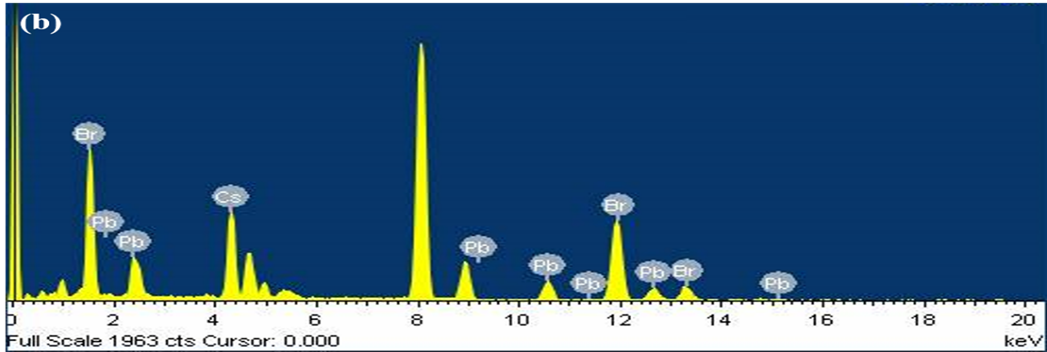
**

**Figure S6.** Energy Dispersive X-ray spectrometer analysis of CsPbBr_3_-loaded nanogels.


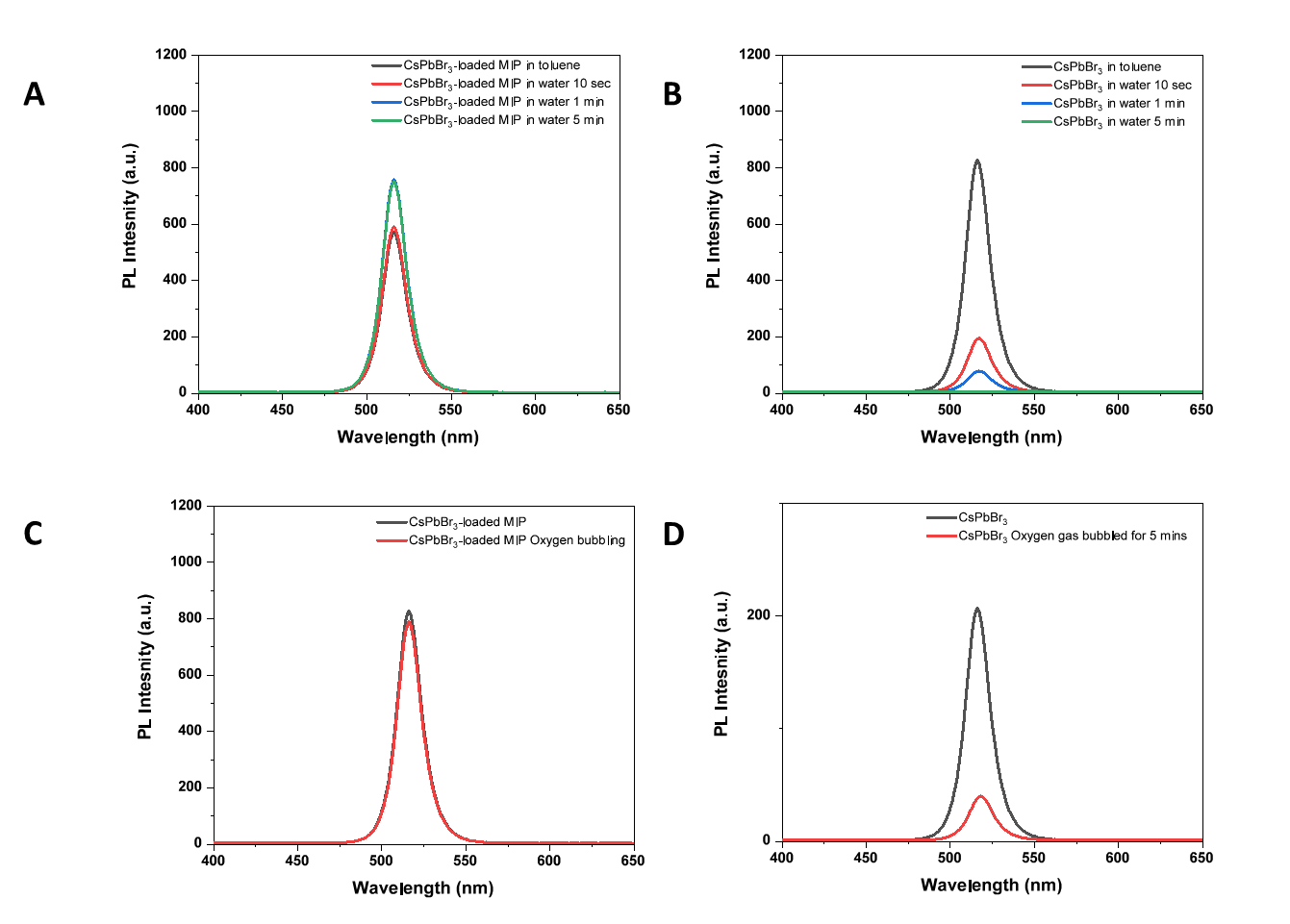


**Figure S7.**The stability studies of CsPbBr_3_ nanoparticles loaded in MIP nanogels towards water and oxygen. The water stability of CsPbBr_3_-loaded MIP nanogel (A), the water stability of bulk CsPbBr_3_ nanoparticles (B), the oxygen stability of CsPbBr_3_-loaded MIP nanogel (C), and the oxygen stability of bulk CsPbBr_3_ nanoparticles (D).

**
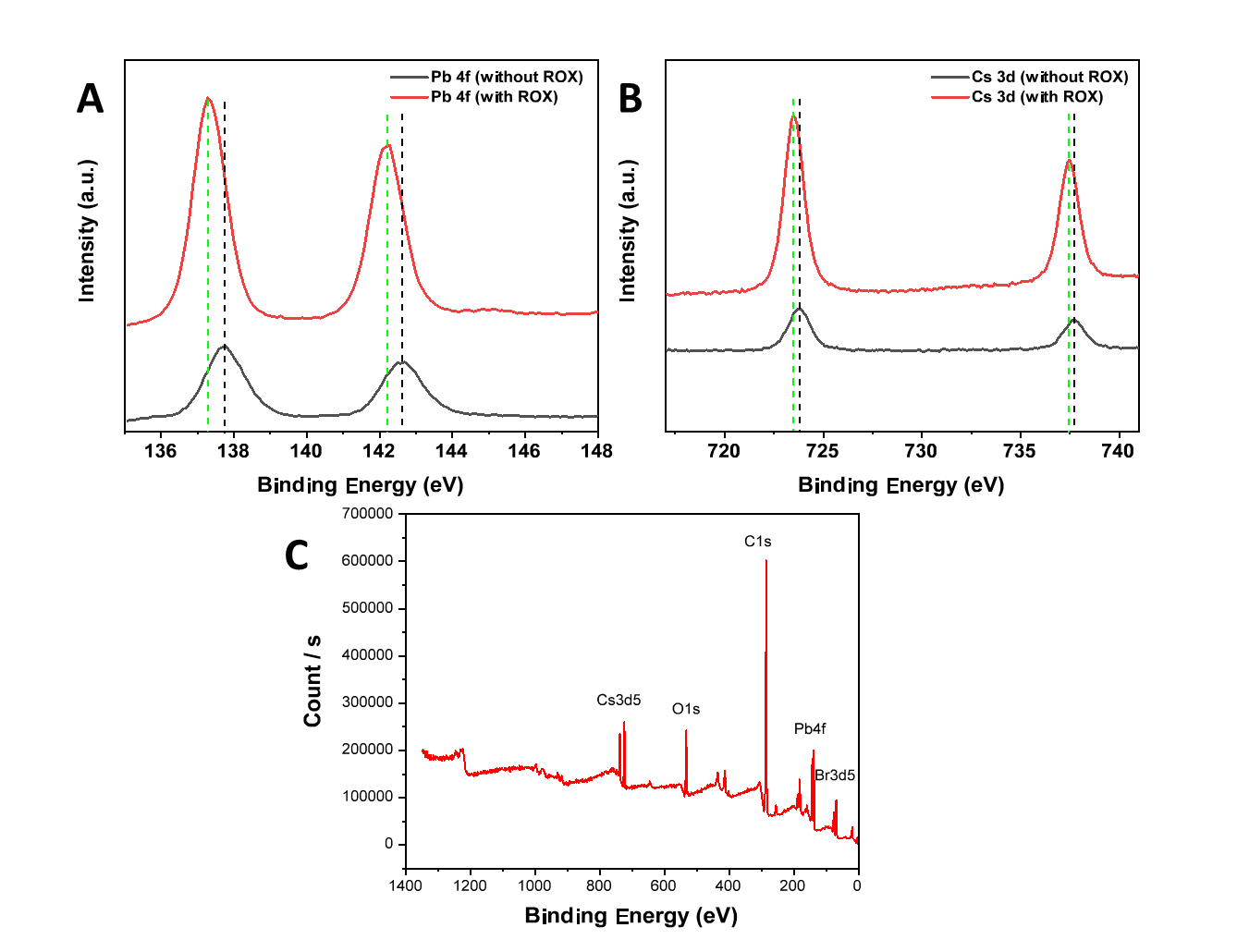
**

**Figure S8.**XPS analysis of perovskite-loaded MIP nanogels before and after ROX treatment. (A) high resolution Pb 4f scan XPS spectra, (B) high resolution Cs 3d scan XPS spectra, and (C) survey scan XPS spectrum of perovskite-loaded MIP nanogels.
